# Supplementary figures and images for: Bacteroides fragilis Promotes Mesenchymal Subtype in Colorectal Cancer
Source: Cancers (Basel). 2025 Nov 28;17(23):3822. doi: 10.3390/cancers17233822 (PMC12691386; doi:10.3390/cancers17233822)

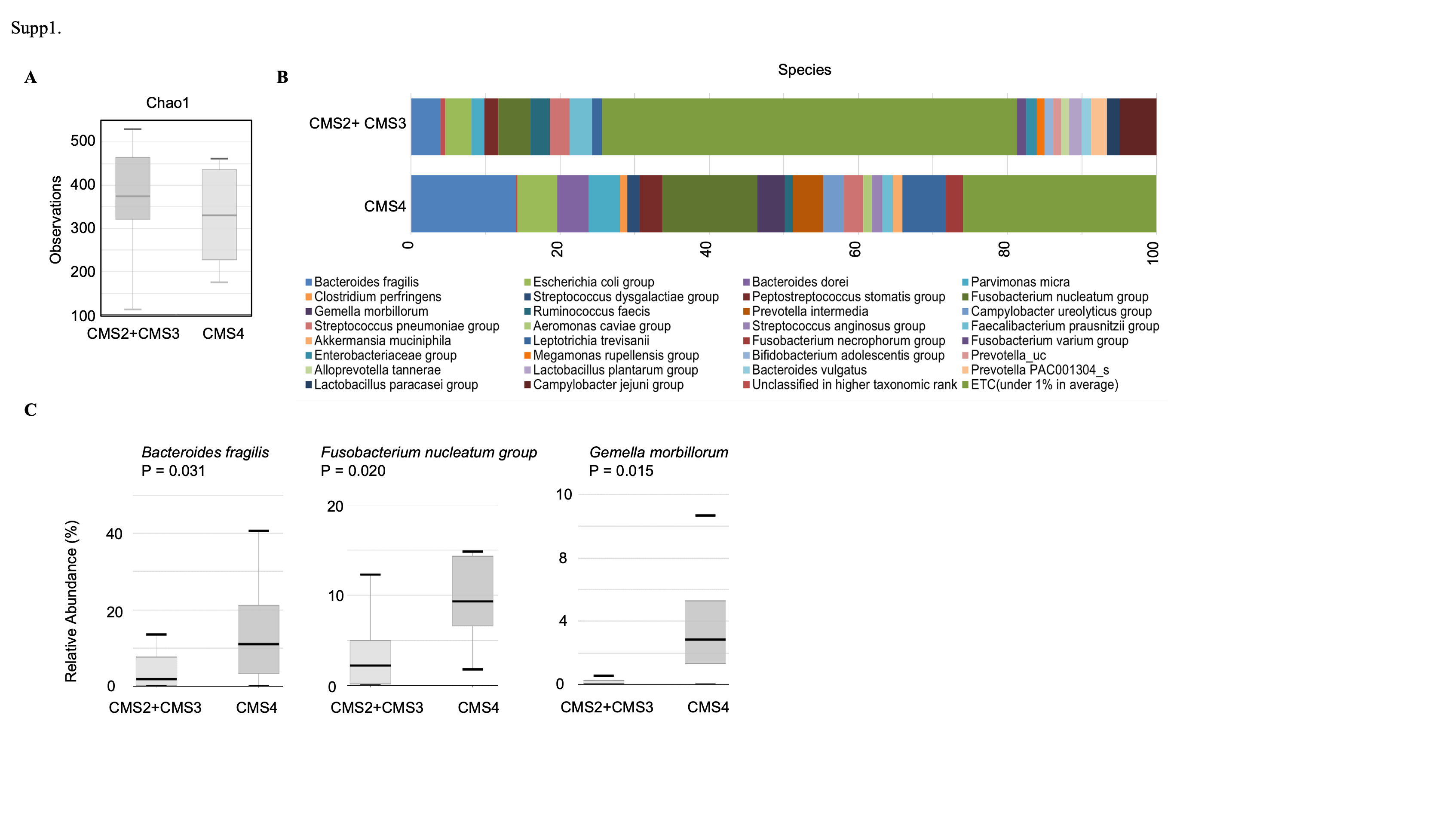

Supplement: Supplementary file 1 [file cancers-17-03822-s001.zip › Supplementary Figure 1.tiff]

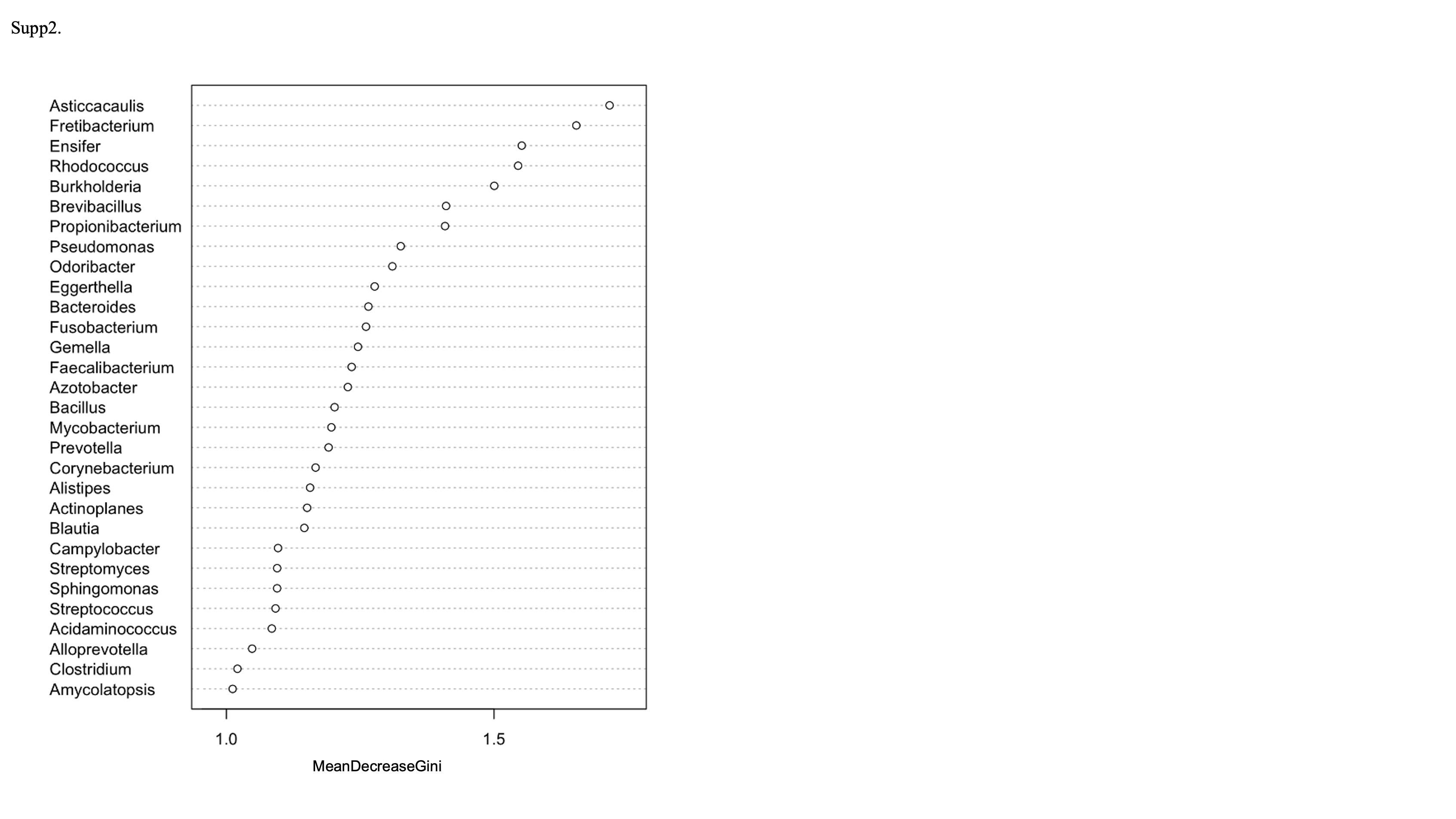

Supplement: Supplementary file 1 [file cancers-17-03822-s001.zip › Supplementary Figure 2.tiff]

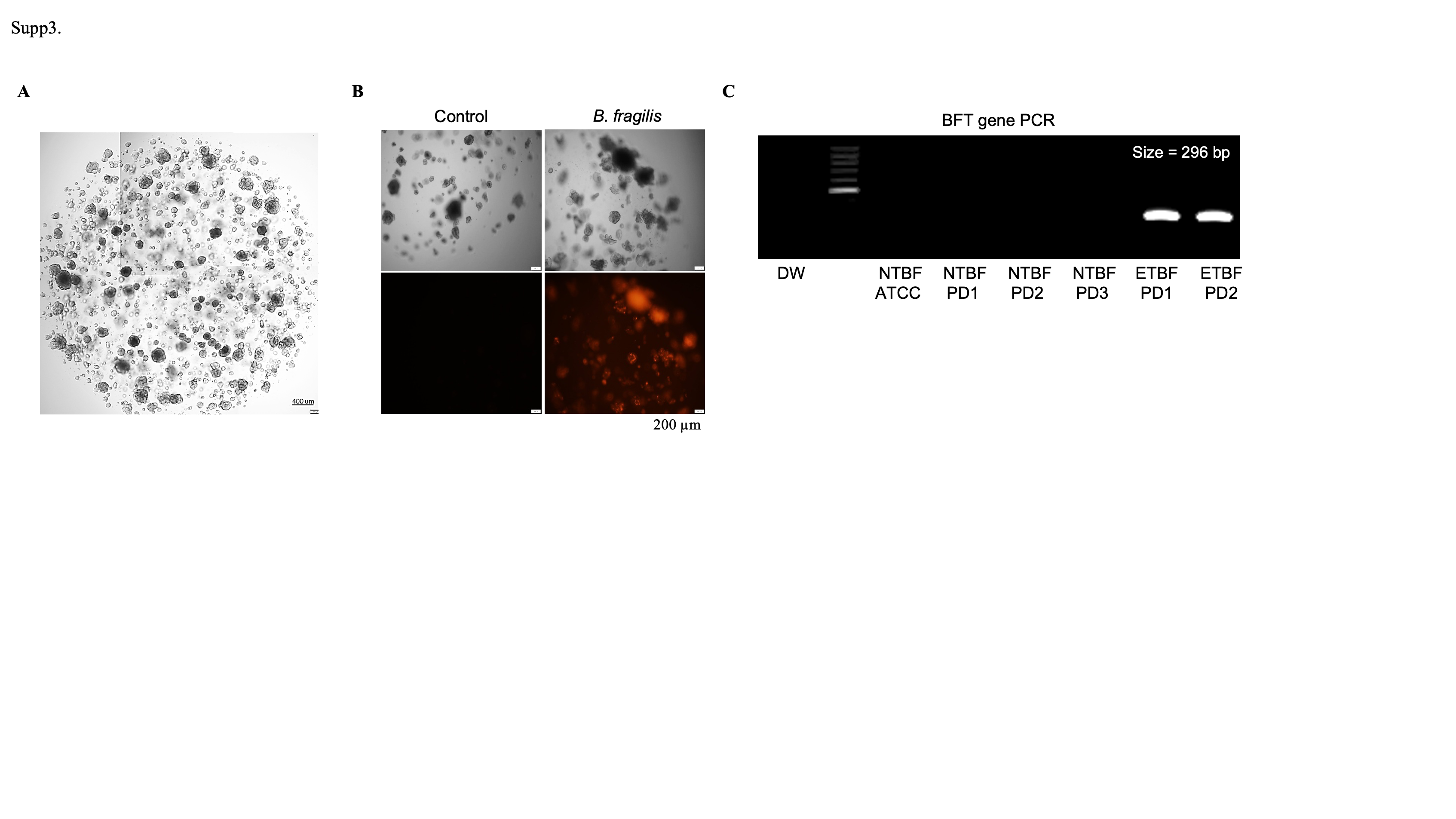

Supplement: Supplementary file 1 [file cancers-17-03822-s001.zip › Supplementary Figure 3.tiff]

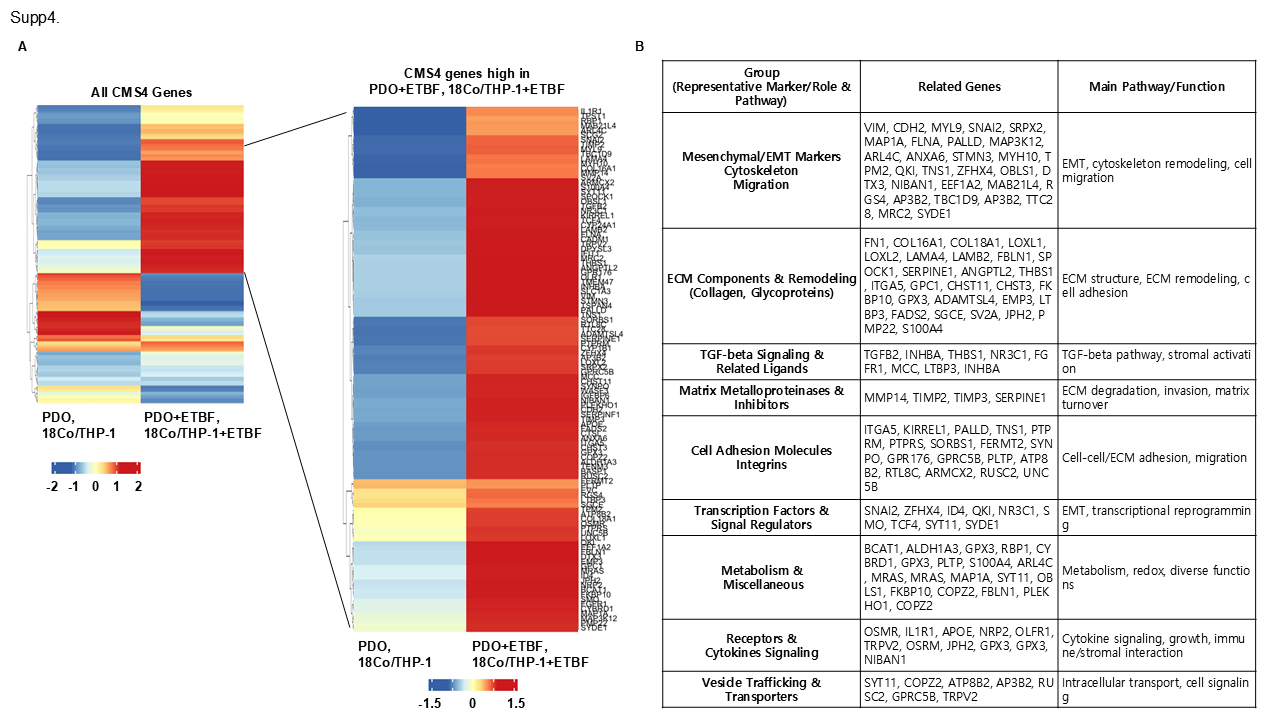

Supplement: Supplementary file 1 [file cancers-17-03822-s001.zip › Supplementary Figure 4.tif]

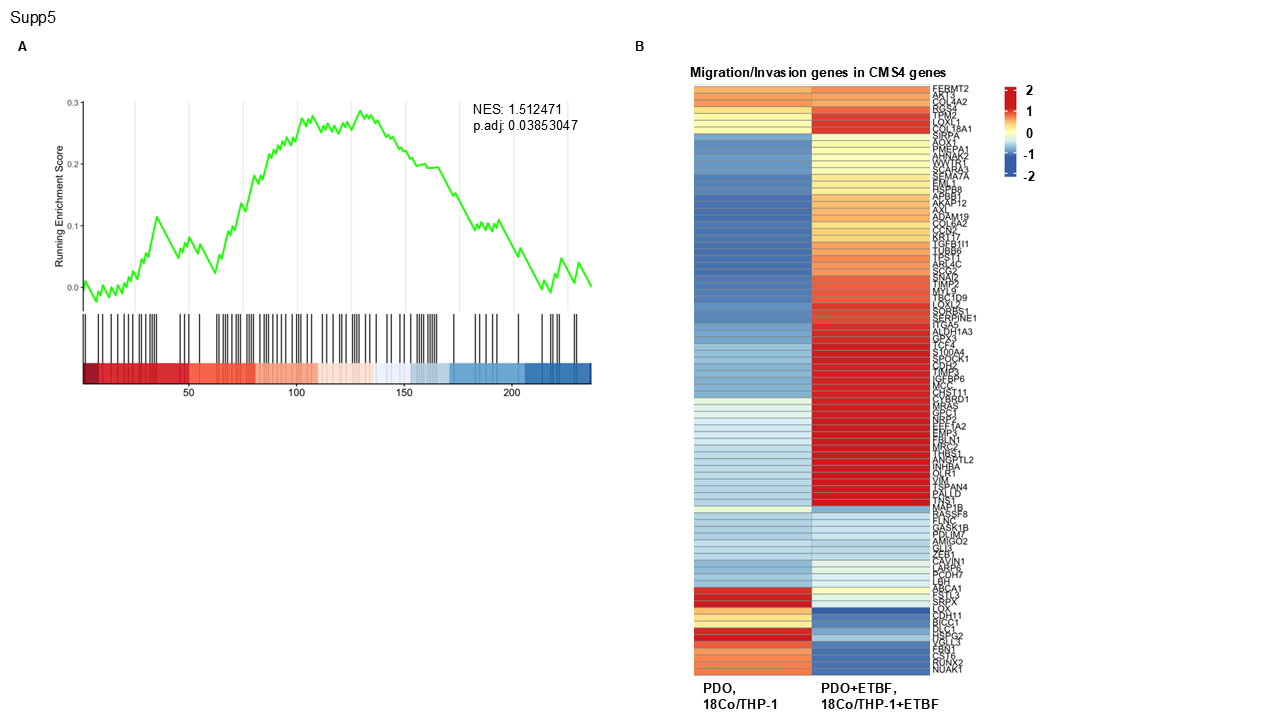

Supplement: Supplementary file 1 [file cancers-17-03822-s001.zip › Supplementary Figure 5.tif]
